# Time at surgery during menstrual cycle and menopause affects pS2 but not cathepsin D levels in breast cancer

**DOI:** 10.1038/sj.bjc.6690145

**Published:** 1999-02

**Authors:** P Pujol, J-P Daurès, J-P Brouillet, T Maudelonde, H Rochefort, J Grenier

**Affiliations:** Service de Biologie Cellulaire, Centre Hospitalier Universitaire de Montpellier-Nîmes, 271, Av G Giraud, Montpellier, 34295, France; Institut Universitaire de Recherche clinique, Centre Hospitalier Universitaire de Montpellier-Nîmes, 271, Av G Giraud, Montpellier, 34295, France; Centre de Lutte Contre le Cancer de Montpellier, Parc Euromédecine, Montpellier, 34000, France

**Keywords:** breast cancer, pS2, cathepsin D, menstrual cycle, menopause

## Abstract

Many studies have addressed the clinical value of pS2 as a marker of hormone responsiveness and of cathepsin D (Cath D) as a prognostic factor in breast cancer. Because pS2 and Cath D are both oestrogen induced in human breast cancer cell lines, we studied the influence of the menstrual cycle phase and menopausal status at the time of surgery on the levels of these proteins in breast cancer. A population of 1750 patients with breast cancer, including 339 women in menstrual cycle, was analysed. Tumoral Cath D and pS2 were measured by radioimmunoassay. Serum oestradiol (E2), progesterone (Pg), follicle-stimulating hormone (FSH) and luteinizing hormone (LH) levels at the day of surgery were used to define the hormonal phase in premenopausal women. There was a trend towards a higher mean pS2 level in the follicular phase compared with the luteal phase (17 ng mg^−1^and 11 ng mg^−1^respectively, *P*= 0.09). Mean pS2 was lower in menopausal patients than in women with cycle (8 ng mg^−1^and 14 ng mg^−1^respectively, *P*= 0.0001). No differences in mean Cath D level were observed between the different phases of the menstrual cycle, or between pre- and post-menopausal women. In the overall population, pS2 was slightly positively associated with E2 and Pg levels and negatively associated with FSH and LH, probably reflecting the link between pS2 and menopausal status. In premenopausal women, no association was found between pS2 and E2, Pg, FSH or LH levels. There were no correlations between Cath D level and circulating hormone levels in the overall population. However, in the subgroup of premenopausal women with ER-positive (ER+) tumours, E2 was slightly associated with both pS2 and Cath D, consistent with oestrogen induction of these proteins in ER+ breast cancer cell lines. There are changes in pS2 level in breast cancer throughout the menstrual cycle and menopause. This suggests that the choice of the pS2 cut-off level should take the hormonal status at the time of surgery into account. In contrast, the level of Cath D is unrelated to the menstrual cycle and menopausal status. 1999 Cancer Research Campaign


British Journal of Cancer (1999) **79**(5/6), 909–914
© 1999 Cancer Research Campaign
Article no. bjoc.1998.0145

### Time at surgery during menstrual cycle and menopause affects pS2 but not cathepsin D levels in breast cancer

**P Pujol[1], J-P Daurès[2], J-P Brouillet[1], T Maudelonde[1], H Rochefort[1] and J Grenier[3]**

[1]Service de Biologie Cellulaire, and [2]Institut Universitaire de Recherche clinique, Centre Hospitalier Universitaire de Montpellier-Nîmes, 271, Av G Giraud, 34295 Montpellier, France; [3]Centre de Lutte Contre le Cancer de Montpellier, Parc Euromédecine, 34000 Montpellier, France

**Summary** Many studies have addressed the clinical value of pS2 as a marker of hormone responsiveness and of cathepsin D (Cath D) as a prognostic factor in breast cancer. Because pS2 and Cath D are both oestrogen induced in human breast cancer cell lines, we studied the influence of the menstrual cycle phase and menopausal status at the time of surgery on the levels of these proteins in breast cancer. A population of 1750 patients with breast cancer, including 339 women in menstrual cycle, was analysed. Tumoral Cath D and pS2 were measured by radioimmunoassay. Serum oestradiol (E2), progesterone (Pg), follicle-stimulating hormone (FSH) and luteinizing hormone (LH) levels at the day of surgery were used to define the hormonal phase in premenopausal women. There was a trend towards a higher mean pS2 level in the follicular phase compared with the luteal phase (17 ng $mg^{-1}$ and 11 ng $mg^{-1}$ respectively, $P = 0.09$). Mean pS2 was lower in menopausal patients than in women with cycle (8 ng $mg^{-1}$ and 14 ng $mg^{-1}$ respectively, $P = 0.0001$). No differences in mean Cath D level were observed between the different phases of the menstrual cycle, or between pre- and post-menopausal women. In the overall population, pS2 was slightly positively associated with E2 and Pg levels and negatively associated with FSH and LH, probably reflecting the link between pS2 and menopausal status. In premenopausal women, no association was found between pS2 and E2, Pg, FSH or LH levels. There were no correlations between Cath D level and circulating hormone levels in the overall population. However, in the subgroup of premenopausal women with ER-positive (ER+) tumours, E2 was slightly associated with both pS2 and Cath D, consistent with oestrogen induction of these proteins in ER+ breast cancer cell lines. There are changes in pS2 level in breast cancer throughout the menstrual cycle and menopause. This suggests that the choice of the pS2 cut-off level should take the hormonal status at the time of surgery into account. In contrast, the level of Cath D is unrelated to the menstrual cycle and menopausal status.

**Keywords:** breast cancer; pS2; cathepsin D; menstrual cycle; menopause

Oestrogen receptor (ER) and progesterone receptor (PgR) status are determined in breast cancer cytosol to predict both the response to endocrine therapy (Osborne et al, 1980) and the likelihood of recurrence (Clark et al, 1988). However, only 50–70% of ER-positive (ER+) tumours respond to anti-hormone therapy, and steroid receptor status is a relatively short-term prognostic factor. Recent studies have investigated the clinical value of pS2 as an additional marker of hormone response (Henry et al, 1989; Schwartz et al, 1991; Spyratos et al, 1994) and of cathepsin D (Cath D) as a prognostic factor in breast cancer (Tandon et al, 1990; Rochefort et al, 1992; Pujol et al, 1993).

There is currently considerable interest in the time of surgery according to the phase of the menstrual cycle in terms of tumour biology (Badwe et al, 1995; Oliver et al, 1995; Holdaway et al, 1997) and prognosis (Senie et al, 1991; Fentiman et al, 1994; Veronesi et al, 1994; Mohr et al, 1996). Some studies suggested that ER levels in human breast cancer underwent changes throughout the menstrual cycle, with an overall trend towards a higher ER level in the follicular phase (Coradini et al, 1984; Holli et al, 1995, Pujol et al, 1998).

*Received 4 March 1998*
*Revised 26 June 1998*
*Accepted 27 July 1998*

*Correspondence to*: P Pujol, Service de Biologie Cellulaire, Hôpital Arnaud de Villeneuve, Centre Hospitalier Universitaire de Montpellier, 271, Av G. Giraud, 34295 Montpellier, France

Cath D is an ubiquitous lysosomal proteinase. Its overexpression in breast cancer tissues has been correlated to poor prognosis, independently of other prognostic factors (Rochefort et al, 1992; Ferrandina et al, 1997). In contrast, a high pS2 level is an indicator of good prognosis and of hormone sensitivity, and is generally correlated to steroid receptor positivity (Foekens et al, 1990). Both pS2 and Cath D proteins have been shown to be oestrogen induced in ER+ breast cancer cell lines (Westley et al, 1980; Masiakowski et al, 1982). Cath D and pS2 might, thus, be regulated throughout the menstrual cycle in breast tumours.

The possible variations of pS2 and Cath D according to the hormonal state raises important questions on the clinical interpretation of these markers in terms of prognosis or hormone dependency, particularly whether it is appropriate to use the same cut-off throughout the reproductive life and menopause. We studied relationships between pS2, Cath D and the hormonal phase, as determined by levels of circulating hormones, in a large population of breast cancer patients.

### PATIENTS AND METHODS

### Patients

Patients with primary breast cancer (1750) were included in a monocentric prospective study from 1988 to 1994 (Cancer Centre of Montpellier, France). All the patients have circulating hormone analyses from a blood sample obtained on the day of surgery. Oral consent was obtained for all patients. Exclusion criteria were

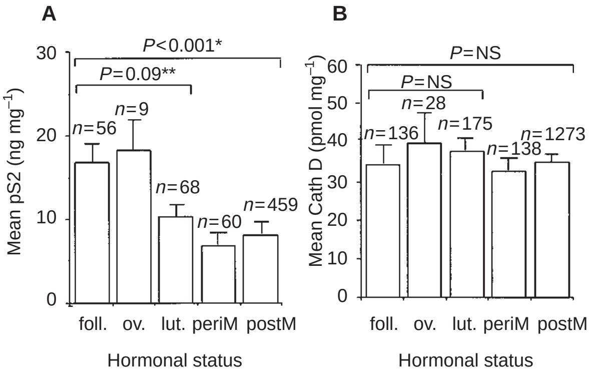

**Figure 1** pS2 and Cath D levels according to the menstrual cycle phase and menopause. Hormonal statuses were determined according to the levels of circulating hormones as described in Patients and methods. foll, follicular phase; ov, ovulatory period; lut, luteal phase; periM, premenopause; postM, post menopause. Bars represent standard errors of the means. *Multiple test comparison (Kruskall–Wallis non-parametric Anova test). **Follicular vs luteal phase. NS, non-significant. (**A**) Mean pS2 level according to the hormonal status. (**B**) Mean Cath D level according to the hormonal status

**Table 1** Correlations between Cath D, pS2 and circulating hormones. (**A**) Overall population; (**B**) premenopausal women

**A**

| | E2 | Pg | FSH | LH |
|---|---|---|---|---|
| Cath D | –0.01* | –0.01 | –0.01 | –0.01 |
| (*n* = 1750) | NS | NS | NS | NS |
| pS2 | 0.15 | 0.13 | –0.16 | –0.10 |
| (*n* = 652) | *0.0002* | *0.001* | *<0.0001* | *0.01* |

**B**

| | E2 | Pg | FSH | LH |
|---|---|---|---|---|
| Cath D | 0.11 | 0.02 | –0.03 | 0.03 |
| (*n* = 339) | *0.04* | NS | NS | NS |
| pS2 | 0.10 | –0.10 | –0.02 | 0.16 |
| (*n* = 133) | NS | NS | NS | NS |

*Spearman rank correlation coefficient. *P*-value is indicated in italic; NS, not significant.

preoperative radiotherapy, neoadjuvant chemotherapy or tamoxifen therapy, use of oral contraceptives less than 1 month before surgery, hormone replacement therapy and pregnancy.

Hormonal status was defined as follows: premenopausal, patients with regular menstrual cycle ($n$ = 339); menopausal, patients with the last regular menses occurring more than 2 years earlier (postmenopausal, $n$ = 1273) or less than 2 years earlier (perimenopausal, $n$ = 138). The hormonal phase of the menstrual cycle in premenopausal patients was determined according to levels of circulating hormones on the day of surgery, including E2 and Pg, follicle-stimulating hormone (FSH) and luteinizing hormone (LH). Women with progesterone values exceeding 2.5 ng ml$^{-1}$ were considered to be in the luteal phase of the cycle ($n$ = 175). The ovulatory period was defined on the basis of both high LH (>10 IU l$^{-1}$) and oestradiol (> 100 pg ml$^{-1}$) values ($n$ = 28). The remaining premenopausal women were classified in the follicular phase ($n$ = 136). The precise date of the last menstrual period was available in 240 premenopausal patients.

### Cytosolic assays

ER and PgR were assayed using the dextran-coated charcoal method (Korenman et al, 1969) with [$^3$H]oestradiol and [$^3$H]progesterone (specific activity, 87 Ci mmol$^{-1}$, NEN, Paris, France). Protein concentration was assayed using the Lowry technique (Lowry et al, 1951). Quality control included both internal controls and EORTC standards. ER or PgR levels of 10 fmol mg$^{-1}$ protein or more were considered positive. Total Cath D and pS2 were measured using immunoradiometric assays (ELSA-cathD and ELSA-pS2 kit, respectively, CIS Bio-Industries, Gif-sur-Yvette, France). Both Cath D and pS2 assays were routinely and prospectively performed in our laboratory. Cath D level was available in all 1750 patients, whereas pS2 level, assayed from 1992 to the end of the study, was performed in a subgroup of the last 652 consecutive patients.

### Circulating hormones

Circulating hormones were assessed routinely in the laboratory of our Cancer Centre from a blood sample obtained on the day of surgery. Sera analyses were performed weekly, blinded of clinical information. E2 and Pg were measured using two commercially available radioimmunoassay kits, ESTR-US-CT and PROG-CTRIA (CIS). LH and FSH were assessed using immunoradiometric [$^{125}$I]hLH Coatria and [$^{125}$I]hFSH Coatria kits, respectively (Biomérieux, Marcy-l'Etoile, France). Oestrogen and progesterone levels are given in pg ml$^{-1}$ and ng ml$^{-1}$ respectively. FSH and LH levels are given in mU ml$^{-1}$.

### Statistical analysis

The Mann–Whitney test or the Kruskall–Wallis test were used to compare non-Gaussian quantitative parameters. Chi-squared or Fisher tests were used to compare receptor positivity frequencies. Correlations between quantitative parameters were analysed with Spearman's test. Multiple tests comparison were performed using the Bonferroni method. The significance level ($P$-value) was set at 0.05.

### RESULTS

### pS2 and Cath D according to the menstrual cycle phase and menopause

The mean pS2 level was higher in the follicular than in the luteal phases (16.8 ng mg$^{-1}$ and 10.9 ng mg$^{-1}$ respectively, Figure 1A), although the difference was not significant ($P$ = 0.09). No changes in Cath D were observed between the different phases of the menstrual cycle (Figure 1B).

To study the effect of menopausal status on pS2 and Cath D levels, the follicular, ovulatory and luteal phases were grouped (premenopausal women), and compared with the group of both peri- and post-menopausal women. The mean pS2 level was higher in premenopausal women than in menopausal women (14.4 ng mg$^{-1}$ and 8.1 ng mg$^{-1}$ respectively, $P$ = 0.0001), whereas no differences in Cath D levels were noted between premenopause and menopause.

 *© Cancer Research Campaign 1999*

**Table 2** Characteristics of patients according to ER status

| Parameter (mean) | ER– (*n* = 751) | ER+ (*n* = 999) | *P*-value |
|---|---|---|---|
| Cath D (pmol mg$^{-1}$) | 29 | 43 | <0.001 |
| pS2 (ng mg$^{-1}$) | 7[a] | 19[b] | <0.0001 |
| RP (fmol mg$^{-1}$) | 39 | 190 | <0.001 |
| E2 (pg ml$^{-1}$) | 81 | 71 | NS |
| Pg (ng ml$^{-1}$) | 2.7 | 2.0 | 0.04 |
| Hormonal status | | | |
| Follicular | 58 (43) | 78 (57) | 0.03* |
| Ovulatory | 13 (46) | 15 (54) | |
| Luteal | 96 (55) | 79 (45) | |
| Menopausal | 584 (41) | 827 (59) | NS** |

Mean values. Numbers in parentheses are percentages. [a]*n* = 234; [b]*n* = 418.
*Chi-squared for trend between follicular, ovulatory and luteal phases.
**Chi-squared for trend between all subgroups.

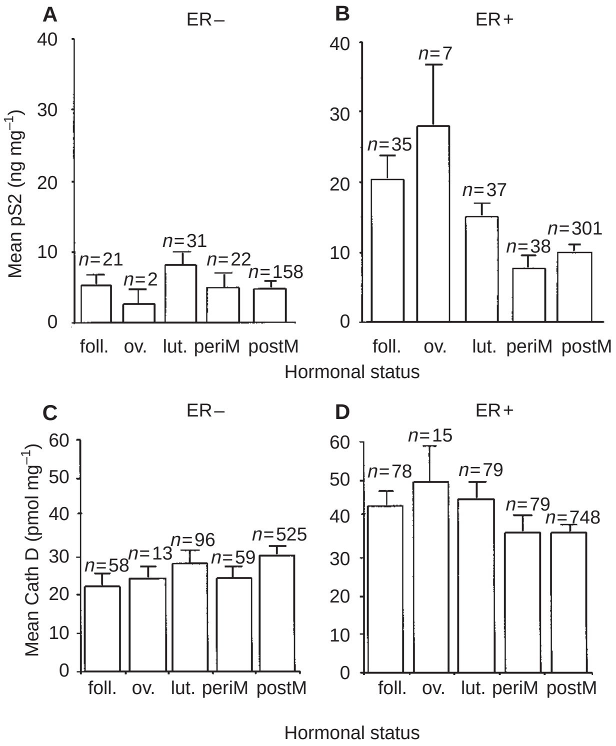

**Figure 2** Mean pS2 and Cath D levels according to the ER status. (**A**) Mean pS2 in ER– tumours. (**B**) Mean pS2 in ER+ tumours. (**C**) Mean Cath D in ER– tumours. (**D**) Mean Cath D in ER+ tumours

### Correlations between pS2, Cath D and circulating hormones

In the overall population, pS2 was positively correlated with E2 and Pg levels and negatively correlated with FSH and LH (Table 1A). However, these associations between pS2 and circulating hormones levels, although statistically significant using Spearman rank correlation (<0.05), were weak as shown by the regression coefficient ($r$). In premenopausal women, no associations were found between pS2 and circulating hormone levels (Table 1B).

There was no correlation between Cath D and levels of E2, Pg, FSH and LH in the overall population (Table 1A). In premenopausal women, Cath D was weakly associated with E2 (Table 1B).

### pS2, Cath D and circulating hormones according to ER status

Because pS2 and Cath D proteins can be oestrogen induced via the ER in breast cancer cell lines, we stratified the population of premenopausal women according to ER status (Table 2). Mean Cath D and pS2 levels were higher in ER+ than in ER– tumours ($P < 0.001$ for both). The mean pS2 and Cath D levels according to the hormonal phase in both ER+ and ER– tumours are shown in Figure 2.

ER positivity decreased during the menstrual cycle ($P = 0.03$, Table 2). Pg and E2 were slightly higher in ER– than in ER+ tumours. In the subgroup of premenopausal women with ER+ tumours, E2 was slightly correlated with both pS2 and Cath D ($r = 0.23$ for both, data not shown).

### Characteristics of patients according to pS2 and Cath D status

Table 3 shows the characteristics of patients according to pS2 status as defined by a cut-off value of 2.5 ng mg$^{-1}$ (median value). pS2 status was positively related to ER, PgR and Cath D levels and influenced by the hormonal status. When the median Cath D value was used as a cut-off value (26 pmol mg$^{-1}$), Cath D was positively associated with both ER and PgR ($P < 0.0001$ for both, Table 4). pS2 was higher in the group with Cath D levels greater than the cut-off level ($P = 0.01$). Cath D status was not related to the hormonal status nor to circulating sex hormones.

### Correlations between pS2, Cath D and steroid receptors

In the overall population, there was a correlation between pS2 and Cath D, but the regression coefficient was low ($r = 0.14$, $P < 0.001$). Cath D was weakly associated with both ER and PgR ($r = 0.26$ and $r = 0.22$ respectively; $P < 0.0001$ for both). pS2 was also weakly associated with both ER and PgR ($r = 0.24$ and $r = 0.40$ respectively; $P < 0.0001$ for both). In the subgroup of premenopausal women, these correlations between all cytosolic proteins were also noted (data not shown).

### Histopathological grade

Seven hundred and thirty-nine patients were analysed to correlate Scraff Bloom and Richardson grade (SBR) with pS2, Cath D, ER, PgR, E2, Pg and hormonal status. pS2 was slightly negatively correlated with SBR grade ($P = 0.03$ respectively, Table 3), whereas Cath D was positively correlated with SBR grade ($P < 0.0001$, Table 4). The percentage of ER-positive tumours was inversely associated with SBR grade (73% in grade I, 69% in grade II and 51% in grade III, $P < 0.001$, data not shown). The percentage of PgR-positive tumours was also lower in grade III

© Cancer Research Campaign 1999

**Table 3** Characteristics of patients according to pS2 status

| | pS2 | | |
|---|---|---|---|
| | < 2.5 ng mg⁻¹ ($n$ = 323) | ≥ 2.5 ng mg⁻¹ ($n$ = 329) | $P$-value |
| ER | | | |
| Mean | 38.4 | 91.3 | <0.0001 |
| ER+ | 170 (41) | 248 (49) | |
| ER– | 153 (65) | 81 (35) | <0.0001 |
| PgR | | | |
| Mean | 72.4 | 130.7 | <0.0001 |
| PgR+ | 150 (35) | 276 (65) | |
| PgR– | 173 (77) | 53 (23) | <0.0001 |
| Cath D | | | |
| Mean | 8.4 | 15.2 | 0.01 |
| SBR[a] | | | |
| I | 25 (37) | 43 (63) | |
| II | 137 (46) | 162 (54) | |
| III | 85 (52) | 78 (48) | 0.03 |
| Hormonal status | | | |
| Follicular | 18 (32) | 38 (68) | NS* |
| Ovulatory | 2 (22) | 7 (78) | |
| Luteal | 24 (36) | 44 (64) | |
| Menopausal | 279 (54) | 240 (46) | <0.001** |

[a]$n$ = 530. *Chi-squared for trends between follicular, ovulatory and luteal phases. **Chi-squared for trends between all subgroups.

**Table 4** Characteristics of patients according to Cath D status

| | Cathepsin D | | |
|---|---|---|---|
| | < 26 pmol mg⁻¹ ($n$ = 855) | ≥ 26 pmol mg⁻¹ ($n$ = 895) | $P$-value |
| ER | | | |
| Mean | 42 | 73 | <0.01 |
| ER+ | 414 (41) | 585 (59) | |
| ER– | 441 (59) | 310 (41) | <0.0001 |
| PgR | | | |
| Mean | 64 | 111 | <0.01 |
| PgR+ | 488 (44) | 622 (56) | |
| PgR– | 367 (57) | 273 (43) | <0.0001 |
| pS2[a] | | | |
| Mean | 8.4 | 15.2 | 0.01 |
| SBR[b] | | | |
| I | 55 (57) | 42 (43) | |
| II | 211 (54) | 183 (46) | |
| III | 105 (42) | 143 (58) | <0.01 |
| Hormonal status | | | |
| Follicular | 65 (48) | 71 (52) | NS* |
| Ovulatory | 10 (36) | 18 (64) | |
| Luteal | 87 (50) | 88 (50) | |
| Menopausal | 693 (49) | 718 (51) | NS** |

[a]$n$ = 652; [b]$n$ = 739. *Chi-squared for trends between follicular, ovulatory and luteal phases. **Chi-squared for trends between all subgroups

(56%) than in grade I (64%) or II (68%, $P$ = 0.02). E2, Pg, FSH, LH and hormonal status were not related to SBR.

### DISCUSSION

Cath D and pS2 have recently been analysed as markers of prognosis or hormone dependency in breast cancer (Henry et al, 1989; Foekens et al, 1990; Tandon et al, 1990; Schwartz et al, 1991; Rochefort et al, 1992; Pujol et al, 1993; Spyratos et al, 1994). Therefore, determinations of these markers at surgery could theoretically be proposed to guide clinicians in adjuvant therapy. Although there is growing interest in the effect of the menstrual cycle at time of surgery on the breast cancer prognosis, little is known on the influence of the hormonal milieu at surgery on these factors. We found that pS2, but not Cath D level, is affected by the menstrual cycle phase and menopausal status at the time of surgery.

Cath D is secreted under the influence of oestrogen in ER+ breast cancer cell lines, but is also constitutively expressed in oestrogen unresponsive breast cancer cell lines (Westley and Rochefort, 1980). In ER+ premenopausal women, we found a weak association between Cath D and E2, suggesting that E2 could in vivo stimulate Cath D expression by breast cancer cells. This is consistent with previous data showing that tamoxifen increases the Cath D level in ER+ patients, probably by acting through its partial agonist oestrogenic effect (Maudelonde et al, 1994). However, we found no change in Cath D expression during the menstrual cycle and menopause, indicating that this marker could be interpreted in clinics independently of the hormonal status.

In contrast, we found a decreased level of pS2 in the luteal phase compared with the follicular phase, and in premenopausal women compared with menopausal women. In the overall population, pS2 was weakly positively correlated with FSH and LH levels and negatively to $E_2$ and Pg. This is probably because of the correlation between pS2 and menopausal status that was noted in our study as in previous reports (Foekens et al, 1990; Predine et al, 1992; Spyratos et al, 1994) because, in premenopause, no correlations were found between E2, Pg, gonadotrophin and pS2. However, in the subset of ER+ premenopausal women, pS2 was correlated with the E2 level, suggesting that pS2 expression, like Cath D, could be increased by E2 in some ER+ breast tumours.

As reported in previous studies, we noted a correlation between pS2 and ER and PgR (Foekens et al, 1990; Predine et al, 1992; Spyratos et al, 1994). There was also a weak association of Cath D with both ER and PgR, as already found in several studies (Cazin et al, 1993; Gion et al, 1995), but not all (Granata et al, 1991). This study also confirmed the association of high Cath D level with SBR grade II and III previously reported (Cazin et al, 1993; Gion et al, 1995), as well as its correlation to pS2 (Gion et al, 1995).

To our knowledge, there is only one other published study on the variation of pS2 and Cath D protein levels during the menstrual phase and menopause (Khan et al, 1997). This study, analysing pS2 and Cath D expression in 69 premenopausal women, also noted no variation of Cath D throughout the cycle. However, the authors found an increased pS2 level in the luteal phase contrasting with our results. This discrepancy may be explained by the difference in methods of pS2 and Cath D determinations [immunohistochemistry in the study by Khan et al (1997) and radioimmunoassay in the present study], and the definition of the hormonal phases [menstrual history in the study by Khan et al (1997) and hormone levels in this report]. At the mRNA level, Saad et al (1998) recently found no significant differences in Cath D expression throughout the menstrual cycle.

*© Cancer Research Campaign 1999*

Changing levels of pS2 throughout the menstrual cycle and menopause may be taken into account to more accurately predict the hormone sensitivity and prognosis of a tumour. In this study, the median value of pS2 (2.5 ng $ml^{-1}$) was arbitrarily chosen as the cut-off level. This threshold value is lower than the cut-off used in some prognostic studies using the same radioimmunoassay, ranging from 4 ng $dl^{-1}$ to 11 ng $dl^{-1}$ (Foekens et al, 1990; Gion et al, 1993). However, when the mean value of pS2 (9.4 ng $dl^{-1}$) was set as a cut-off level, the increased level of pS2-positive tumours in premenopausal women compared with post-menopausal women was also observed (40% vs 22%, $P < 0.0001$, data not shown). Further clinical studies with available follow-up are needed to define which threshold is more reliable in predicting hormone sensitivity and prognosis according to the hormonal status.

The findings of several studies assessing the effect of the menstrual cycle phase at the time of surgery on prognosis have been conflicting. Several studies (Badwe et al, 1991; Senie et al, 1991; Veronesi et al, 1994) and a meta-analysis (Fentiman et al, 1994) showed an increased overall survival rate in women with breast cancer who had undergone surgery in the luteal phase. Other studies, however, failed to demonstrate such a significant effect of timing of surgery on prognosis (Powles et al, 1991; Rageth et al, 1991). Because no correlations were found between Cath D levels and the menstrual phase, it is unlikely that hormonal induction of total Cath D could explain the prognostic differences according to the time of surgery. It is noteworthy that the effect of the menstrual phase on steroid receptors and pS2 could potentially modify the prediction of hormone sensitivity, and thus be a bias for the choice of adjuvant hormone therapy. The population of the present study is being prospectively analysed in order to study the prognostic value of circulating sex hormone levels and the phase of the menstrual cycle.

In conclusion, there are changes in pS2, but not Cath D levels, in breast cancer during the menstrual cycle and menopause. This suggests that interpretations of hormone dependency on the basis of pS2 level should take the hormonal status at the time of surgery into account. In contrast, it may not be necessary to consider the hormonal status of the patients when using Cath D levels as a marker of poor prognosis. Further clinical studies are needed to define the appropriate pS2 cut-off level to be used relative to the hormonal status at the time of surgery to improve predictive information on prognosis and hormone responsiveness.

### ACKNOWLEDGEMENT

This work was supported by the ‘Centre Hospitalier Universitaire de Montpellier’ and the ‘Centre Régional de Lutte Contre le Cancer de Montpellier’.

### REFERENCES

Badwe RA, Gregory WM, Chaudary MA, Richards MA, Bentley AE, Rubens RD and Fentiman IS (1991) Timing of surgery during menstrual cycle and survival of premenopausal women with operable breast cancer. *Lancet* **337**: 1261–1264

Badwe RA, Bettelheim R, Millis RR, Gregory W, Richards MA and Fentiman IS (1995) Cyclical tumour variations in premenopausal women with early breast cancer. *Eur J Cancer* **13**: 2181–2184

Cazin JL, Gosselin P, Boniface B and Demaille A (1993) Expression of PS2 and cathepsin D in breast cancer. *Int J Oncol* **2**: 1081–1084

Clark GM and McGuire WL (1988) Steroid receptors and other prognostic factors in primary breast cancer. *Semin Oncol* **15**: 20–25

Coradini D, Cappelleti V, Miodini P, Ronchi E, Scavone G and Di Fronzo G (1984) Variations in estrogen and progesterone receptor content in premenopausal breast cancer patients throughout the menstrual cycle. *Tumori* **70**: 339–344

Fentiman IS, Gregory WM and Richards MA (1994) Effect of menstrual phase on surgical treatment of breast cancer. *Lancet* **344**: 402

Ferrandina G, Scambia G, Benedetti Panici P, Mancuso S and Messori A (1997) Relationship between cathepsin D content and disease-free survival in node-negative breast cancer patients: a meta-analysis. *Br J Cancer* **76**: 661–666

Foekens JA, Rio MC, Seguin P, Van Putten WLJ, Fauque J, Nap M, Klijn JGM and Chambon P (1990) Prediction of relapse and survival in breast cancer patients by pS2 protein status. *Cancer Res* **50**: 3832–3837

Gion M, Mione R, Dittadi R, Romanelli M, Capitanio G, Friede U, Barbazza R, Visona A and Dante S (1995) Relationship between cathepsin D and other pathological and biological parameters in 1752 patients with primary breast cancer. *Eur J Cancer* **31A**: 671–677

Granata G, Coradini D, Cappeletti V and Difronzo G (1991) Prognostic relevance of cathepsin D vs oestrogen receptors in node-negative breast cancer. *Eur J Cancer* **27**: 970–972

Henry JA, Nicholson S, Hannesy C, Lennard T, May F and Westley B (1989) Expression of the oestrogen related pNR-2 mRNA in human breast cancer: relation to oestrogen receptor mRNA levels and response to tamoxifen therapy. *Br J Cancer* **63**: 32–38

Holdaway IM, Mason BH, Lethaby AE, Harman JE, France JT and Knox BS (1997) Characteristics of the menstrual cycle at the time of surgery for breast cancer. *Br J Cancer* **75**: 413–416

Holli K, Isola J and Hakama M (1995) Prognostic effect of timing of operation in relation to menstrual phase of breast cancer patient – fact or fallacy. *Br J Cancer* **71**: 124–127

Khan SA, Gonchoroff NJ and Miller LE (1997) Expression of pS2, c-erbB-2, and cathepsin D during the menstrual cycle in human breast cancers. *Ann Surg Oncol* **4**: 462–469

Korenman SG (1969) Comparative binding of estrogens and its relation to estrogenic potency. *Steroids* **13**: 163–178

Lowry OH, Roseborough N, Farr L and Randall RJ (1951) Protein measurement with the Folin phenol reagent. *J Biol Chem* **193**: 265–275

Masiakowski P, Breathnach R, Bloch J, Gannon F, Krust A and Chambon P (1982) Cloning of cDNA sequences of hormone-related genes from the MCF-7 human breast cancer cell line. *Nucleic Acids Res* **10**: 7895–7903

Maudelonde T, Escot C, Pujol P, Rouanet P, Defrenne A, Brouillet JP and Rochefort H (1994) In vivo stimulation by tamoxifen of cathepsin D mRNA level in breast cancer. *Eur J Cancer* **30A**: 2049–2053

Mohr PE, Wang DY, Gregory WM, Richards MA and Fentiman IS (1996) Serum progesterone and prognosis in operable breast cancer. *Br J Cancer* **73**: 1552–1555

Oliver DJ and Ingram DM (1995) Timing of surgery during the menstrual cycle for breast cancer: possible role of growth factors. *Eur J Cancer* **3**: 325–328

Osborne CK, Yochmowitz MG, Knight WA and McGuire WL (1980) The value of estrogen and progesterone receptors in the treatment of breast cancer. *Cancer* **36**: 2884–2888

Pink JJ and Jordan VC (1996) Models of estrogen receptor regulation by estrogens and antiestrogens in breast cancer cell lines. *Cancer Res* **56**: 2321–2330

Powles TJ, Ashley SE, Nash AG, Tidy A, Gazet JC and Ford HT (1991) Menstrual surgical timing (letter). *Lancet* **337**: 1604

Predine J, Spyratos F, Prud’homme JF, Andrieu C, Hacene K, Brunet M, Pallud C and Milgrom E (1992) Enzyme-linked immunosorbent assay of pS2 in breast cancers benign tumors, and normal breast tissues. *Cancer* **69**: 2116–2123

Pujol P, Maudelonde T, Daures JP, Rouanet P, Brouillet JP, Pujol H and Rochefort H (1993) A prospective study of the prognostic value of cathepsin D levels in breast cancer cytosol. *Cancer* **71**: 2006–2012

Pujol P, Daures JP, Thezenas S, Guilleux F, Rouanet P and Grenier J (1998) Changing estrogen and progesterone receptor patterns in breast cancer during the menstrual cycle and menopause. *Cancer* **83**: 698–705

Rageth JC, Wyss P, Unger C and Hochuli E (1991) Timing of breast cancer surgery within the menstrual cycle: influence on lymph-node involvement, receptor status, postoperative metastatic spread and local recurrence. *Ann Oncol* **2**: 269–272

Rochefort H (1992) Biological and clinical significance of cathepsin D in breast cancer. *Acta Oncol* **31**: 125–130

Saad Z, Bramwell VHC, Wilson SM, O’Malley FP, Jeacock J and Chambers AF (1998) Expression of genes that contribute to proliferative and metastatic ability in breast cancer resected during various menstrual phases. *Lancet* **351**: 1170–1173

© Cancer Research Campaign 1999 

Schwartz L, Koerner F, Edgerton SM, Sawicka JM, Rio MC, Bellocq JP, Chambon P and Thor D (1991) pS2 expression and response to hormonal therapy in patients with advanced breast cancer. *Cancer Res* **51**: 624–628
Senie RT, Rosen PP, Rhodes P and Lesser ML (1991) Timing of breast cancer excision during the menstrual cycle influences the duration of disease-free survival. *Ann Intern Med* **115**: 337–342
Spyratos F, Andrieu C, Hacene K, Chambon P and Rio MC (1994) pS2 and response to adjuvant hormone therapy in primary breast cancer. *Br J Cancer* **69**: 394–397
Tandon AK, Clark GM, Chamness GC, Chirgwin JM and McGuire WL (1990) Cathepsin D and prognosis in breast cancer. *N Engl J Med* **322**: 297–302
Veronesi U, Luini A, Mariani L, Del Vecchio M, Alvez D, Andreoli C, Giacobone A, Merson M, Pacetti G, Raselli R and Saccozzi R (1994) Effect of menstrual phase on surgical treatment of breast cancer. *Lancet* **343**: 1544–1546
Westley BR and Rochefort H (1980) A secreted glycoprotein induced by estrogen in human breast cancer cell line. *Cell* **20**: 352–362

*© Cancer Research Campaign 1999*
